# NegotiAge—An AI‐Based Caregiver Negotiation Training: Results From the Multiphase Optimization Strategy (MOST) Trial

**DOI:** 10.1111/jgs.70546

**Published:** 2026-06-17

**Authors:** Vanessa Ramirez‐Zohfeld, Alaine Murawski, Celie Jobin, Charles Olvera, Johnathan Mell, Jeanne Brett, Jonathan Gratch, Marianne Tschoe, Angela F. Pfammatter, Lee A. Lindquist

**Affiliations:** ^1^ Division of Geriatrics Northwestern University, Feinberg School of Medicine Chicago Illinois USA; ^2^ University of Central Florida Orlando Florida USA; ^3^ Center on Dispute Resolutions, Northwestern University, Kellogg School of Business Chicago Illinois USA; ^4^ Vanderbilt University Nashville Tennessee USA; ^5^ University of Tennessee Knoxville Knoxville Tennessee USA

**Keywords:** artificial intelligence, conflict resolution, family caregivers, negotiation

## Abstract

**Background:**

Family caregivers experience conflicts in caring for people with Alzheimer's dementia (PWD; e.g., siblings disagreeing, advocating with providers, PWD refusing assistance). Subsequently, family caregivers experience frustration, burden, and stress. Furthermore, these caregivers rarely receive formal conflict resolution training to ameliorate these concerns. We developed NegotiAge—an artificial intelligence (AI)‐based negotiation training intervention that teaches caregivers how to resolve conflicts and provides the opportunity to practice negotiating with online avatars in real‐world conflicts. We sought to optimize the potency and test the early effect of NegotiAge on family caregivers following the multiphase optimization strategy (MOST).

**Participants, Design, Setting:**

National randomized factorial trial of NegotiAge with family caregivers of PWD (> 65 years). Caregivers were randomized to one of eight conditions and variables/outcomes assessed at baseline and 1 month following NegotiAge. The mixed methods project used dependent sample *t*‐tests (quantitative) and constant comparative analysis (qualitative).

**Results:**

Across 23 states, 125 family caregivers (mean‐age 54 years, 77% female) completed the study. Participants experienced significantly less caregiver burden (−3.24, *p* < 0.002) and negative affect (−2.1, *p* < 0.0001) between baseline and 1 month following. Those receiving two or more exercises experienced further significant burden reduction (−5.28, *p* < 0.04). Qualitative analyses supported these findings: “There was a time when my mother was crying. Because of the training I was able to calm her.”; “When we have a conflict, I focus on the interest of both of us–helped tremendously.” In the month following, 54% (*n* = 67) of participants experienced conflicts and 74% (*n* = 93) applied skills learned. Among utilizers, there was a significant decrease in forcing behaviors (−1.24, *p* < 0.0001).

**Conclusions:**

NegotiAge, an AI‐based negotiation training intervention, significantly reduced burden and improved negative affect among family caregivers. Engaging in more negotiation exercises further reduced burden. NegotiAge has the potential to improve the lives of caregivers and PWD. Further testing is needed to determine the direct impact on older adults and long‐term effects.

**Trial Registration:**

ClinicalTrials.gov identifier: NCT04837937

## Summary

- Key Points
  ○ Family caregivers experience conflicts when interacting with and advocating for their older adults with Alzheimer's dementia (PWD). NegotiAge is an AI‐based online negotiation training program that teaches family caregivers how to resolve conflicts and enables them to practice negotiating with avatars receiving real time feedback on how to improve their skills.
  ○ Using the multiphase optimization strategy (MOST) framework, we conducted a national randomized factorial trial with four different NegotiAge exercises with family caregivers of PWD (*n* = 125, 23 states) with data collection at baseline, immediately, and 1 month following completion of NegotiAge.
  ○ One month after completing NegotiAge, family caregivers experienced significantly less burden (*p* < 0.005), less negative affect (*p* < 0.05), and qualitative analysis revealed the family caregivers had life‐changing experiences with their PWD following training.
- Why Does This Paper Matter?
  ○ Family caregivers are frequently unprepared to handle conflicts that occur when working with and advocating for their older adult loved ones. Participating in NegotiAge, an online AI‐based negotiation program, reduced caregiver burden and improved negative affect after 1 month following use. Scalable interventions for caregivers that reduce burden are efficient (around 45 min) and can be completed on the users' schedule are vital to aiding family caregivers and improving the care of PWD.

## 1 Introduction

Approximately 37.1 million people provide unpaid care to adults aged 65 or older, encompassing both family and friend caregivers [1, 2, 3]. Family caregivers of older people with Alzheimer's dementia (PWD) face unique care challenges that frequently escalate and entail coordination with other family caregivers, multiple healthcare providers, private, and community‐based service agencies, as well as insurers [4, 5, 6]. Family caregivers are often ill‐prepared for their roles and provide care with little or no training [7]. As a result, caregiving for others is associated with declines in physical and mental health, and health‐related quality of life [8, 9, 10, 11]. Caregiver well‐being also influences PWD health by impacting their emotional affect, interfering with how well caregivers assess their pain and other symptoms, and influencing long‐term medical decision‐making for the patient (e.g., nursing home/long‐term care placement) [12].

Prior research has shown that caregivers frequently encounter conflicts related to the care of the PWD, leading to both emotional and psychological challenges. Conflicts may revolve around differing perceptions of the older adult's needs, decision‐making authority, and perceived fairness of caregiving responsibilities [13]. Caregivers also encounter conflict when taking on the role of patient advocate in negotiating optimal healthcare for their loved ones. As advocates for their PWD, caregivers encounter conflicts related to communication with healthcare providers, fragmented transitional care, unaddressed clinical concerns, and financial considerations (e.g., insurance) [6].

**FIGURE 1:**
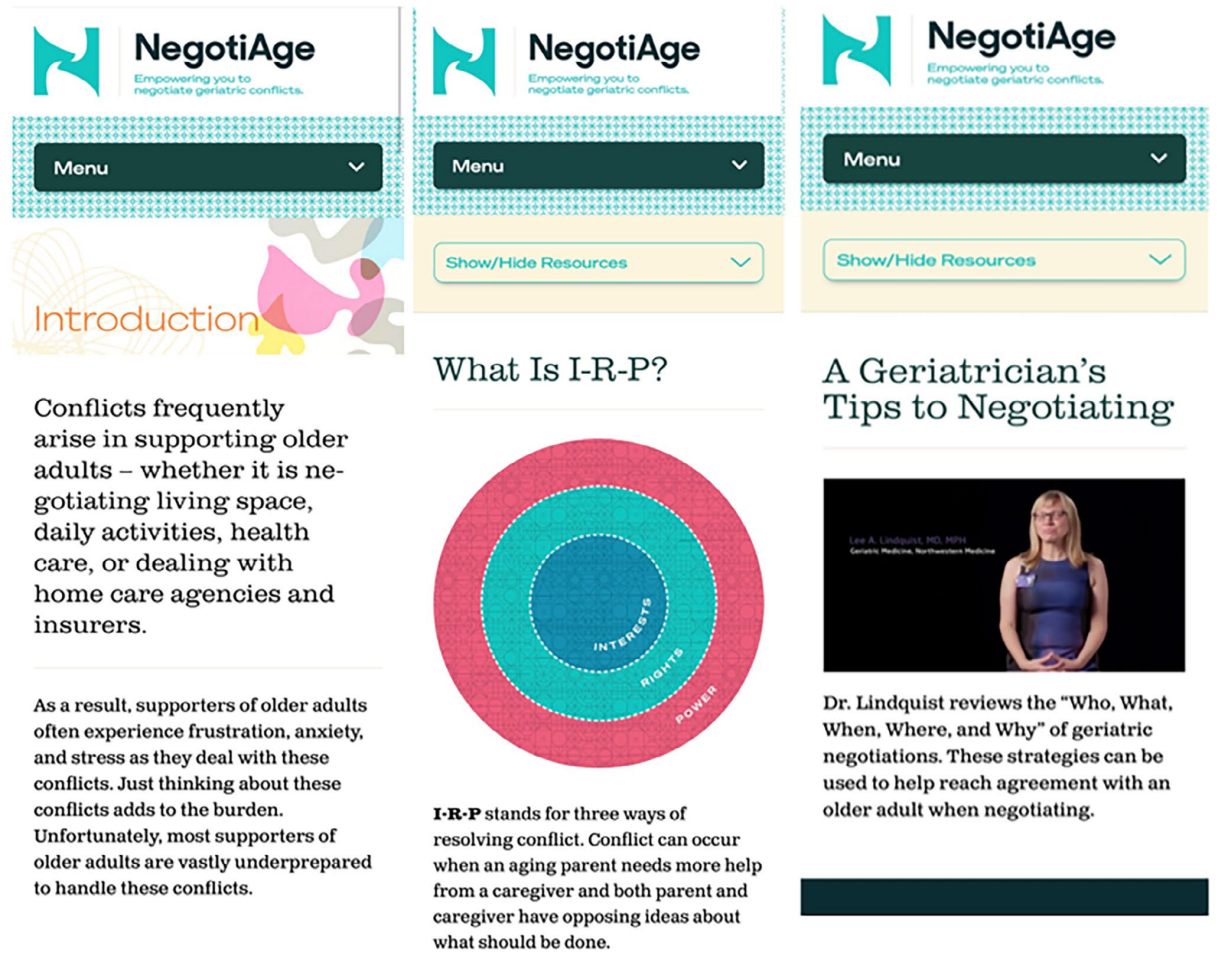
NegotiAge.

To support caregivers who experience conflicts, we developed an artificial intelligence (AI)‐based negotiation training program, NegotiAge (Figure 1). Tailored specifically to family caregivers of PWD, NegotiAge is a 45 min online intervention, that provides short videos (3–7 min) on negotiation (e.g., how to negotiate, framework of negotiation, geriatric tactics), resources (e.g., planning worksheets, readings), and the ability to practice negotiating with AI‐based avatars who respond with human emotions and responses. NegotiAge allows participants to communicate with an AI agent that dynamically chooses from a dataset of over 1000 dialogue lines derived from a panel of family caregivers, geriatricians, social workers, nurses, and older adults. The development of NegotiAge has been previously published, and it utilizes the IAGO Agent platform as a foundational technology [14, 15].

The multiphase optimization strategy (MOST) is a newer method for building and evaluating e‐health interventions. MOST entails several phases: (1) Preparation phase, in which intervention components are identified for potential selection in an intervention package; (2) Optimization phase, in which the selected components are fine‐tuned, and questions such as optimal component dosage for efficiency are investigated; and (3) Evaluation phase, in which the optimized intervention, consisting of selected components, is evaluated in a standard large scale confirmatory trial [16].

Through the MOST, we sought to optimize and test NegotiAge on family caregivers using a randomized factorial trial. Our objective was to identify the active components of the intervention that positively affect patient‐centered outcomes—such as caregiver well‐being and self‐efficacy—as well as secondary outcomes, including reductions in stress, burden, and anxiety.

## 2 Methods

### 2.1 NegotiAge Intervention

NegotiAge consists of a collection of conflict and dispute resolution instructional resources: multimedia (video) and print materials, as well as an AI‐based conflict negotiation platform [8]. Short videos focus on teaching caregivers how to resolve conflicts using a negotiation framework, communication strategies, and a geriatrician's advice to negotiate with older adults. Text‐based instructional resources include a document to assist family caregivers in planning for a negotiation by setting goals and identifying interests, rights, and power for each person involved in the negotiation. The hands‐on negotiation element of the intervention utilized the Interactive Arbitration Guide Online (IAGO), a platform enabling caregivers to negotiate in real time with avatars who are designed to act like humans exhibiting emotions and irrational behaviors [17, 18].

As shown in Table 1, all study participants were assigned the universal negotiation scenario, featuring an older adult and a family caregiver in conflict over the older adult's reluctance to accept in‐home support for activities of daily living (e.g., household cleaning). In addition, participants could be randomized to up to three additional conflict situation exercises: (1) a disagreement between two family caregivers over the appropriate level of care for the older adult, (2) a conflict between a caregiver and a healthcare provider concerning the physician's recommended care plan for the older adult, and (3) a more challenging version of the universal negotiation scenario. Caregivers sent and received “offers,” using strategies from the didactic learning materials until either mutual agreement was reached, or time expired (7 min). Immediate feedback on the negotiation was generated for participants to help them improve their negotiation skills. All possible NegotiAge exercises can be completed in 45 min.

**TABLE 1:** MOST intervention conditions.

Experimental condition	Negotiation activities, by relationship type conflict			
	Caregiver‐older adult [universal]	Caregiver—sister	Caregiver‐physician	Caregiver‐older adult^a^
1	Yes	No	No	No
2	Yes	No	No	Yes
3	Yes	No	Yes	No
4	Yes	No	Yes	Yes
5	Yes	Yes	No	No
6	Yes	Yes	No	Yes
7	Yes	Yes	Yes	No
8	Yes	Yes	Yes	Yes

^a^ Advanced version of the universal activity.

### 2.2 Participant Recruitment

Participants were recruited nationally through community partners, informational talks, emails bursts, and “snowballing” word of mouth. To be eligible, participants were required to be: (1) age ≥ 21 years, (2) able to read and speak English, (3) currently providing at least 1 h per week caregiving support (e.g., emotional, social, physical, task‐related) to an adult age ≥ 65 years who had cognitive loss (e.g., ≥ 2 on the eight‐item informant interview to differentiate aging and dementia test) [19] (4) involved in decision‐making related to the healthcare and support of the older adult, and (5) have access to a computer with internet and a valid email address. All study procedures were approved by the Institutional Review Board at Northwestern University and registered on clinicaltrials.gov (NCT04837937).

### 2.3 Data Collection

Upon completion of the electronic informed consent, participants were sent a link to the baseline assessment (T1) for completion in REDCap. Following completion of the survey, participants were provided with a study link which included their embedded randomization assignment and gave them access to the NegotiAge intervention and their assigned negotiation scenarios. Participants were asked to complete electronic surveys at 1 week, 1 month, and 3 months post‐intervention completion. The 3 month follow‐up data collection is ongoing and will be reported in future analyses. Participants retained access to NegotiAge (i.e., intervention hosting educational materials, AI negotiation scenarios) throughout the entire 3 month follow‐up period and could revisit their assigned negotiation scenarios.

### 2.4 Measures and Outcomes

Study participants self‐reported sociodemographic information such as age, sex, race, ethnicity, marital status, employment status, education, hours of caregiving, and annual household income. Caregiver outcomes included positive affect and well‐being (Neuro‐QoL), caregiver anxiety (Neuro‐QoL TBI—modified to reflect dementia), caregiver burden (Zarit Burden Interview), physical, mental, and social health (PROMIS) [20, 21, 22]. Specific to negotiation, outcomes were collected on participants' conflict management strategies (DUTCH Test for conflict handling), negotiation knowledge, intervention utilization, and perception.

Participants were also asked whether they utilized the intervention, if they had experienced any conflicts, and if so, described the conflict(s) and whether they applied negotiation strategies, affecting their ability to handle the conflict(s). Feasibility (acceptability and usability) was measured through the System Usability Scale (SUS) and usefulness, satisfaction, and ease of use (USE) questionnaire, which measures a person's perceived usability of the intervention in four dimensions: usefulness, USE, ease of learning, and satisfaction with the intervention [23, 24, 25].

Additional descriptions of the measures as well as what timepoint(s) they were collected are listed in Table S1.

### 2.5 Data Analysis

Descriptive frequencies were produced for participant and older adult care recipient demographics. Primary analyses were conducted using intent‐to‐treat linear mixed effects models accounting for data collected at multiple assessment time points nested within individual participants. For each component, we tested differences in change in outcomes across time, with baseline values as the reference. Thus, effects are modeled as component × time interactions. To assess the effects of the intervention on outcomes, measure scores between baseline (T1) and 1 month following intervention (T3) were converted to t scores and paired *t*‐tests were conducted to evaluate the changes in score between timepoints. Generalized linear mixed modeling was used to assess differences between treatment levels and total assigned treatments. To assess negotiation knowledge change between T1 and T3, we conducted univariate ANOVA to examine means by timepoint and employed dependent‐samples *t*‐tests to assess changes over time. We measured utilization of NegotiAge at 1 month post‐intervention with binary (yes/no) questions regarding participants' utilization of NegotiAge, then qualitative open‐ended self‐reported use of negotiation strategies, whether they experienced any conflicts during this time, and if applicable, any real‐world deployment of negotiation strategies, either with the older adult or in any other aspect of their lives. Responses from the open‐ended questions were analyzed using constant comparative techniques with coders independently creating a preliminary list of themes, meeting to refine themes, and resolving any identified discrepancies through discussion [26, 27].

## 3 Results

**FIGURE 2:**
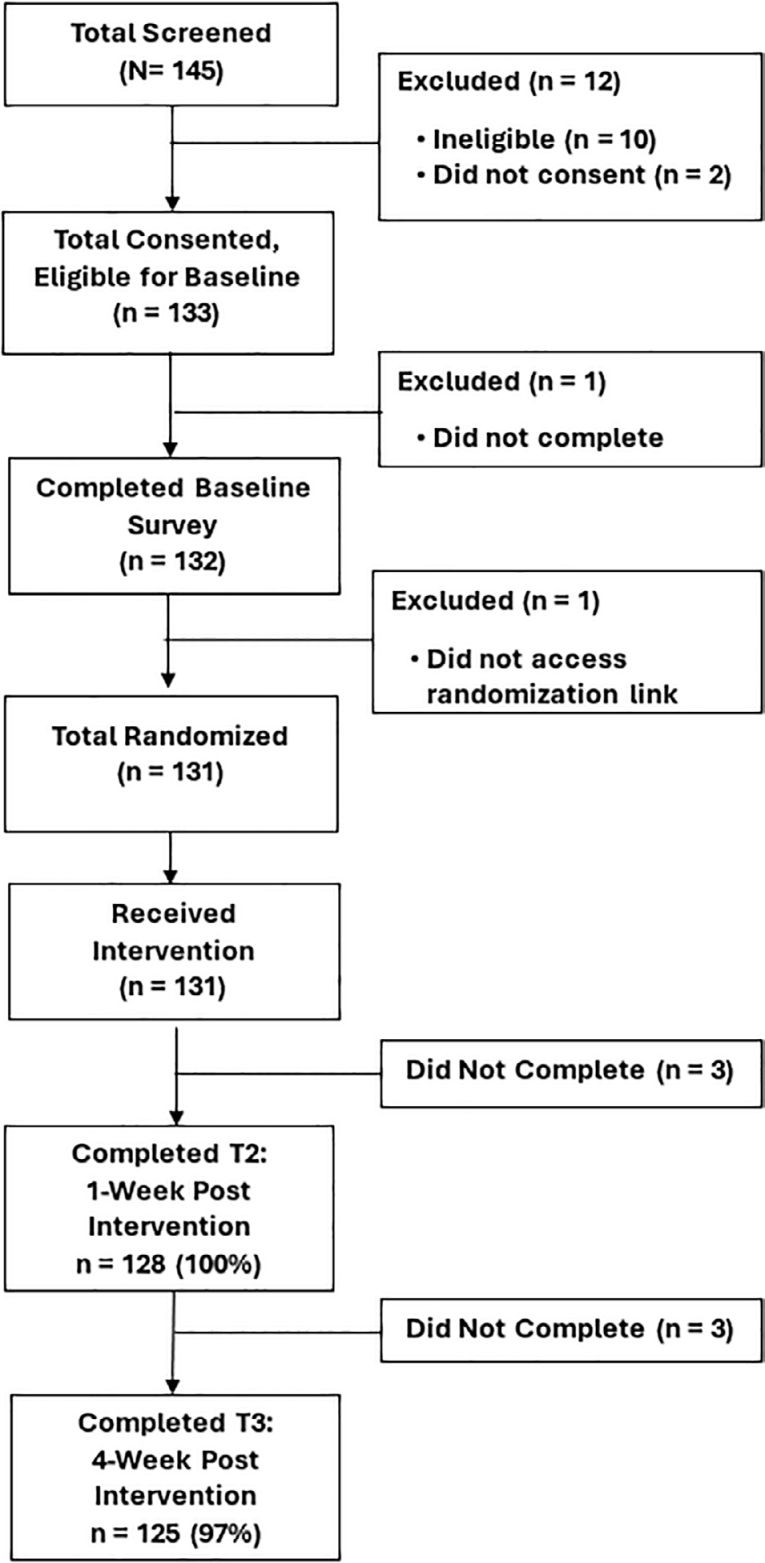
CONSORT diagram.

One hundred and 32 (*N* = 132) family caregivers, across 23 states, completed the baseline T1 survey and 125 completed the 1 month survey (T3) (Figure 2). Participants were an average 53.7 years old (SD = 13.91 years), white (61.4%), female (77.7%), and non‐Hispanic (84.8%). Most caregivers were married (60.6%), employed at least part time (52.3%) and reported an annual household income of less than $100,000 (56.8%) (Table 2).

**TABLE 2:** Participant demographics (*N* = 132).

Characteristics	Mean [SD] or *n* (%)
Age, mean (years)	53.7 [13.9]
Female	102 (77.3)
Regions (23 states)	
Midwest (IL, WI. MO, MI, IN, OH)	56 (42.4)
South (AL, AR, FL, GA, NC, TN, TX)	41 (31.1)
West (CA, AZ, CO, OR, WA)	27 (20.4)
East (CT, MA, MD, NY, VA)	8 (6.1)
Race	
White	81 (61.4)
Black or African American	39 (29.5)
Asian	6 (4.5)
Native Hawaiian or other Pacific Islander	1 (0.8)
Other	3 (2.3)
Prefer not to say	2 (1.5)
Ethnicity	
Hispanic	10 (7.6)
Unknown	10 (7.6)
Marital status	
Never married	18 (13.6)
Married or partnership	88 (66.7)
Divorced	21 (15.9)
Other	5 (3.8)
Annual household income	
< $50,000	34 (25.7)
$50,000 to $100,000	41 (31.1)
> $100,000	43 (32.6)
Prefer not to say	14 (10.6)
Employment status	
Full time	55 (41.7)
Part time	14 (10.6)
Unemployed or unable to work	16 (12.1)
Retired	33 (25.0)
Other	14 (10.6)
Education	
Less than high school	2 (1.5)
High school/GED equivalent	11 (8.3)
Some college, no degree	26 (19.7)
College degree	53 (4.5)
Bachelor's degree	47 (35.6)
Master's degree or higher	40 (30.3)
Lives with older adult with dementia	53 (40.2)
Length of time since starting caregiving	
Less than 1 year	21 (16.0)
Less than 2 years	23 (17.4)
2–5 years	49 (37.1)
More than 5 years	39 (29.5)
Caregiver relation to older adult	
Spouse or partner	16 (12.1)
Adult offspring	68 (51.5)
Other family member	44 (33.3)
Friend	2 (1.5)
Other	2 (1.5)
Emotional support (min 6–max 30)	24.6 [5.56]

From baseline to 1 month follow‐up, significant improvements were observed in caregiver burden and conflict‐handling behavior. Over half of caregivers (62.4%, *n* = 78) reported a reduction in burden which was confirmed by a significant reduction (−3.24, *p* < 0.002) on the Zarit Burden Interview scale. In examining the individual components of NegotiAge, significant burden reduction was observed between participants receiving 1 exercise versus 2 or more exercises (−5.28, *p* < 0.04). There was a significant reduction in negative affect (mean change = −2.1, *t* = 4.1, *p* < 0.0001) as measured on the positive and negative affect schedule (PANAS). Those participants receiving the highest dose (four negotiation exercises) experienced the greatest mean change in negative affect (−4.2, SD 7.3) (Table 3).

**TABLE 3:** Outcome changes between baseline and 1 month with associated *t*‐tests.

Outcome	Baseline mean (SD)	1 month mean (SD)	Mean change (SD)	*t*‐value	*p*
Positive affect & well‐being	54.27 (5.61)	54.88 (5.97)	−0.6 (4.99)	−1.35	0.22
Anxiety	9.39 (3.31)	9.07 (3.01)	−0.36 (2.87)	−1.4	0.16
Caregiver‐specific anxiety	16.02 (5.18)	15.73 (4.82)	−0.4 (3.80)	−1.18	0.24
Burden	37.83 (15.35)	34.59 (14.29)	−3.24 (1.03)	−3.14	< 0.0001^a^
General self‐efficacy	15.53 (2.92)	15.50 (2.92)	−0.022 (2.71)	−0.1	0.74
Fatigue	18.58 (4.97)	17.99 (4.84)	−0.78 (4.52)	−1.92	0.06
Satisfaction with social roles and activities	26.69 (7.12)	26.74 (6.91)	0.10 (4.96)	0.23	0.81
Negative affect	21.78 (7.08)	19.70 (7.28)	−2.1 (5.7)	4.1	< 0.0001^a^
Negotiation knowledge	77.29 (13.66)	80.32 (12.72)	3.09 (13.19)	2.62	0.01*

^a^ Significant (*p* < 0.05).

At 1 month follow‐up, 74% (*n* = 93) of participants stated that they had applied the lessons learned from NegotiAge and 52% (*n* = 65) had engaged in negotiations specific to their older adult care. Among the 93 participants who had utilized the NegotiAge material in real life, there was again a significant reduction in both caregiver burden (mean change = −3.53, *t* = −3.0, *p* = 0.004) and negative affect (mean change = −2.3, *t* = −3.8, *p* = 0.0002), as compared to those who reported they did not apply lessons learned. There were no significant differences in well‐being by treatment package delivered, by time, or by treatment over time. From a feasibility standpoint, the mean score of the USE was 5.47 (SD±1.1) and SUS was 2.94. which reflects good usability and acceptability scores. Between baseline (T1) and 1 month post intervention (T3), 58% (*n* = 72) of participants re‐visited NegotiAge.

Negotiation knowledge score increased significantly between baseline and 1 month post intervention (mean change = 3.09, *t* = 2.62, *p* = 0.01), as did identification of interest statements (mean change = 4.93, *t* = 3.18, *p* < 0.01). This identification corresponds to the material covered in NegotiAge which taught an interest‐rights‐power negotiation framework [28].

Family caregiver conflict management behaviors (e.g., how caregivers respond to conflicts) changed favorably. Between T1 and T3, participants were significantly less likely to avoid conflicts and experienced increased compromising and problem‐solving behaviors as measured by the DUTCH. Participants specifically exhibited significantly more “low forcing” scores (*p* = 0.05). Additionally, among those who had utilized NegotiAge in a negotiation in the 1 month following (*n* = 93), participants experienced a lower forcing the DUTCH (mean = −1.24, SD = 2.96, *t* = −4.04, *p* = 0.0001).

Open‐ended responses regarding positive feedback (e.g., what did you like about NegotiAge) revealed the following themes:

- Useful program content/resources

Videos were very helpful, and I appreciated the supportive tone. In my experience, this kind of information about how to negotiate productively with older adults is hard to find, even with the internet.

The reference cards and videos were very eye opening and helpful, especially understanding the aspect of ‘rights’, how bringing up safety and health concerns can backfire in the negotiation. Learning to recognize and use ‘interests’ was very valuable lesson!

- Ease of use

The virtual scenarios were refreshing and made me more engaged with the content. Easy to navigate.

Virtual participation and instructions are well described. Exercises were easy to follow.

- Innovative AI training methods (game‐like)

I really loved the simulator because it allowed to you to gain some real time practice on common scenarios you may encounter using the negotiation strategies learned.

I did like how the faces changed depending on my response.

Seeing types of responses, one could receive from their loved one or a provider, Seeing the impact of my different statements had on the character, the ability for the “game” to come to a compromise.

- Improved emotional regulation

The range of responses was a helpful reminder of reactions to avoid, even when stressed.

Helped me develop more empathy. Became more aware of how my communication impacted my mother.

Creating calming attitude, creating a positive attitude.

This was a great reminder to acknowledge and respect your loved one's desires. It also reminds me to show empathy to my loved one, even if I may not agree with their decisions.

- Relevance to real life

Realizing that caregiver can utilize negotiation skills was novel. And gave me a fresh perspective to my role.

I appreciate the attention to the difficult dynamics caregivers have to cope with.

Felt very personized considering the scenarios and surveys relate to my life. Gave me insight on how my care can be improved.

Once I found out my mom had dementia I didn't think I would have to negotiate with her as far as her living situation. The negotiation has been helpful in navigating my mom's day‐to‐day living situation. Just simple things have to be negotiated now.

Family caregivers found the training to be rewarding and were eager to utilize what they had learned into their daily routine. One family caregiver stated, Learning about negotiations was ideal, I had no idea how to come across to my dad in a way he would respond to before this. Another participant noted the scenarios helped them navigate caring for a loved one, I appreciate the attention to the difficult dynamics caregivers have to cope with.

Open‐ended responses regarding negative feedback (e.g., what did you dislike about NegotiAge) revealed the following themes:

- Dislike of time limits on each exercise

Felt rushed in time—had to give response in time limit.

Pressure from the timed aspect of the game.

- AI interface

The simulation gives responses very quickly. It was frustrating at first but then I felt it was similar to human dialog.

The AI character would end a thought/statement and then start the next statement that often did not feel related.

- Request for more feedback

Would like more detailed negotiation assessments following each exercise.

Needs feedback round by round as negotiation proceeds. More didactic.

- Too few scenarios

Would have loved more practice samples

I really enjoyed the training and material however maybe there could have been more scenarios in the simulator.

- Technical issues (e.g., stalled dialogue/avatar)

When I made an “offer,” my offer showed up in the “text string” of the negotiation as the other person's response.

Technical issues (*n* < 10 participants) mentioned in the qualitative feedback have since been corrected.

## 4 Discussion

NegotiAge is an AI‐based negotiation training program that was shown to significantly reduce burden and improve conflict resolution communication skills among family caregivers of older adults with dementia. Negotiation knowledge gains, especially those related to interests, rights, and power statements, are notable, given that successful negotiations redirect right or power‐focused arguments to ones that are interest‐based. Many caregivers, 74% (*n* = 93), applied the skills learned from the negotiation training in the month following—both in caring for their older adults and in other areas. Qualitative analysis revealed supporting statements reflecting NegotiAge improved the lives of caregivers and reduced burden. “There was a time when my mother was sad and crying. Because of the training I was able to calm her.” “I consciously thought out my choice of words; I used to give in to what my grandma wanted even when it wasn't in her best interest. Now when we have a conflict, I focus on the interest of both of us and find common ground—helped tremendously.”

A dose effect occurred such that participants who received two or more exercises demonstrated significantly greater proficiency than those who received one exercise. The exercises ranged across a myriad of real‐world opponents—including an agreeable older adult, a challenging older adult, a sibling, and a health care provider. This parallels negotiation teaching in business schools in that the more negotiations one performs, the better the individual becomes at negotiating.

With regards to how NegotiAge impacted negotiation communication, the results of the DUTCH demonstrated a decline in forcing behaviors and improved problem‐solving. By being less forceful and more mutual solution oriented, people communicate with less stress and feel better—a win‐win for both the caregiver and the individual on the receiving end. Qualitative analysis supported these quantitative findings as caregivers were changing their communication behaviors: “Being so intentional is a brain change for me. I am delicate with my words, but this plan required a next‐level care with words that was harder than I expected. Practice, practice, practice …”

The novel AI‐technology which helped caregiver practice negotiating in real time with avatars was found to be feasible, reflecting good usability and acceptability scores. While generating over 1000 lines of dialogue for the avatars was time‐consuming, we chose to use this methodology instead of the chat generative‐based version, to ensure the responses were accurate and to control the movement of the negotiation. IAGO used its own language and response selection engine to pull the most appropriate response for a given reply. This ensured the responses were accurate but leveraged AI to improve the interactivity of the negotiation, which increased overall realism.

Several limitations exist in this study. One limitation is that this study was a randomized factorial trial planned for optimization of the NegotiAge intervention and as such, not powered, like a large scale randomized controlled trial, to detect changes in all the planned outcomes. The self‐reported utilization of the negotiation training in real‐world situations coupled with improved knowledge results may suggest that the trial was underpowered to observe effects on other study outcomes. There were several outcomes that approached significance but may become significant with more participants. Nonetheless, that caregiver burden was significantly lowered by a 45 min intervention is impressive. Measurement of time spent on NegotiAge was also a limitation. Participants were provided with the prescribed individual NegotiAge exercises but could return as much as they wanted over the 1 month follow‐up. We were able to track return use of NegotiAge but detecting the precise amount of return time was challenging due to outliers such as participants who left the screen on (e.g., spending an unrealistic 100 h) or likely showed others the website (e.g., spending < 5 min).

In the future, we hope to conduct a larger scale study of NegotiAge with avatars customized to user needs and powered for effectiveness. The AI‐based technology behind NegotiAge was shown to be feasible for family caregivers to use for training. Further training interventions for family caregivers and other health care providers might leverage this technology to learn asynchronously, when busy individuals have available time. Future research should also endeavor to understand for whom and under what conditions this intervention is most helpful and effective. In conclusion, NegotiAge is an AI‐based online training program that teaches family caregivers the basics of how to negotiate, then enables them to practice their negotiation skills with avatars providing real time responses and feedback on how to improve their skills. Using the MOST framework, we conducted a national randomized factorial trial of four different NegotiAge exercises with family caregivers. At 1 month following completion of NegotiAge, family caregivers experienced significantly less burden, less negative affect, and described real‐world improvement in the care of their loved ones with Alzheimer's disease. NegotiAge is a novel AI‐based negotiation training program that can provide family caregivers with the skills to reduce the burden caused by interpersonal conflicts and potentially improve the care of older adults with Alzheimer's disease.

## Author Contributions

All authors met criteria for authorship by (1) providing substantial intellectual contribution to the study's conception and design (all authors), data acquisition (V.R.‐Z., A.M., C.J.), data analysis (V.R.‐Z., A.M., C.J., C.O., M.T., A.F.P.), and interpretation (all authors); drafting the article or revising it critically for important intellectual content (all authors); and approving the final version to be published (all authors).

## Funding

This work was supported by the National Institute on Aging, (P30AG0599882, R01AG058777, R01AG068421, R01AG0830344).

## Disclosure

The sponsor was not involved in the design, methods, analysis and interpretation of the data, and preparation of the manuscript.

## Conflicts of Interest

The authors declare no conflicts of interest.

## Supporting information


**Table S1:** Description of outcome measures, by timepoint.
