# Supplementary material for: NegotiAge—An AI‐Based Caregiver Negotiation Training: Results From the Multiphase Optimization Strategy (MOST) Trial
Source: J Am Geriatr Soc. 2026 Jun 17;74(7):2032–40. doi: 10.1111/jgs.70546 (PMC13418792; doi:10.1111/jgs.70546)
Supplement: Supplementary file 1 — Table S1: Description of outcome measures, by timepoint. [file JGS-74-2032-s001.pdf]

Table Supplemental. Description of Outcome Measures, by Timepoint

Measure	Description	Scale	Timepoint		
			T1	T2	T3
Patient-Centered Quality of Life and Psychological Health Outcomes					
Neuro-QoL Positive Affect and Well-Being (PAWB)	How caregivers experience feelings of positive affect / well-being.	Frequency: 1 (Never) to 5 (Always)	X		X
PROMIS Anxiety Short Form-4	Anxiety over the past 7 days. Higher scores indicate greater anxiety.		X		X
Neuro-QoL Caregiver v2.0 – TBI-CareQoL Caregiver-Specific Anxiety# – Short	Anxieties that caregivers experience related to health, safety and future well-being of the person Higher scores indicate greater anxiety.		X		X
Zarit Burden Interview (ZBI-22)	Caregiver burden. Higher scores indicate greater caregiver burden.		X		X
PROMIS Self-Efficacy - Short Form	Confidence in one's ability to successfully perform specific tasks.		X		X
PROMIS Fatigue - Short Form 7a	Fatigue and its impact on daily life of caregivers.		X		X
PROMIS Satisfaction with Social Roles - Short Form 8a	Satisfaction with ability to participate in social roles and activities.	1 (Not at All) to 5 (Very Much)	X		X
Conflict and Negotiation Outcomes					
Dutch Test for Conflict Handling (DUTCH)	Conflict management behaviors in the domains of yielding, compromising, forcing, problem-solving, avoiding.	1 (Never) to 5 (Always)	X	X	X
Negotiation Knowledge	Negotiation and conflict resolution knowledge. Higher scores indicate greater negotiation knowledge.	Scoring: 0 to 15	X	X	X
Negotiation Utilization	Self-reported utilization of intervention, application of negotiation strategies.	Binary (yes/no)			X
The Subjective Value Inventory (SVI)	End-user's perceived outcomes of a negotiation by assessing negotiator's feelings about the outcome, process.	1 (Not at All) to 7 (Perfectly)		X	X
Positive and Negative Affect Schedule (PANAS)	Measures positive and negative emotions.	1 (Not at All) to 5 (Extremely)	X	X	X
